# Postoperative Antibiotic Prophylaxis Following Cardiac Implantable Electronic Device Placement

**DOI:** 10.19102/icrm.2019.100804

**Published:** 2019-08-15

**Authors:** Galen M. Kabulski, Amanda Northup, Barbara S. Wiggins

**Affiliations:** ^1^Department of Pharmacy Services, Medical University of South Carolina, Charleston, SC, USA; ^2^College of Pharmacy, Medical University of South Carolina, Charleston, SC, USA; ^3^Department of Medicine, Division of Cardiology, Medical University of South Carolina, Charleston, SC, USA

**Keywords:** Antibiotic, cardiac implantable electronic device, implantable cardioverter defibrillator, permanent pacemaker, prophylaxis

## Abstract

Infections related to cardiac implantable electronic device (CIED) placement are associated with poor clinical outcomes. As such, preprocedural prophylactic antibiotic therapy is indicated for all patients prior to device insertion. However, the available data are less clear on the impact of postprocedural antibiotic therapy on rates of CIED infection when used in addition to preprocedural therapy. This is single-center, retrospective cohort study of 913 patients who underwent CIED-related procedures between October 2010 and August 2014 sought to compare the rate of CIED infections in patients receiving only preprocedural antibiotics with those receiving both preprocedural and postprocedural antibiotics. Univariate analysis was used to detect independent risk factors for CIED infection. After excluding patients receiving concomitant antibiotics for other conditions, those undergoing CIED extraction alone, and those with a lack of follow-up data and/or adequate documentation of clinical encounters, 569 patients were identified for inclusion in the final analysis. The majority of patients who received postprocedural antibiotics received three to five days of therapy, with the most common antibiotic used being cephalexin. There was no statistically significant difference in the incidence of infection between patients who did and did not receive postoperative antibiotics (4.5% versus 6.1%; p = 0.398). In a multivariate analysis, the use of postprocedural antibiotic therapy was not a significant risk factor for infection (adjusted odds ratio: 0.692; 95% confidence interval: 0.314–1.525; p = 0.361). It is therefore reasonable to withhold prescribing postoperative antibiotics in patients following CIED implantation. Individualized risk factor evaluation of patient comorbidities and procedural characteristics may be needed to aid in determining whether postoperative antibiotics are reasonable in different patients. The validity of these findings is contingent on further confirmation via a prospective, randomized clinical trial.

## Introduction

Cardiac implantable electronic devices (CIEDs), including permanent pacemakers (PPMs), implantable cardioverter-defibrillators (ICDs), and chronic resynchronization therapy (CRT) devices, reduce morbidity and mortality in a variety of patient populations.^1–4^ The reported incidence of CIED infection varies from less than 1% to more than 7%, with more recent research suggesting rates of 0.5% to 2.2%.^5^ As the implantation of CIEDs becomes more widespread, the incidence of CIED infection parallels this increase and continues to be a serious complication that clinicians must consider during all phases of CIED care.^6^

The spectrum of CIED infection ranges from moderate examples, such as superficial surgical site inflammation or generator pocket infection, to more severe or even potentially life-threatening ones like endocarditis or bacteremia.^5,7,8^ The more serious CIED infections frequently necessitate hospitalization and the administration of intravenous antibiotics. In some cases, device removal is indicated, which is associated with increased morbidity, including renal failure and the need for intensive care.^6^ Mortality is higher in those requiring device removal as compared with those who do not develop a infection, and this phenomenon may persist for years after device removal.^5,6,9^ These findings signal that the prevention of infection and the minimization of the need for device extraction is of paramount importance in the perioperative setting.

Risk factors for CIED infection are well-documented, with a lack of preprocedural antibiotic prophylaxis^10–14^ and the number of prior CIED procedures a patient has undergone^11,13^ being the most frequently cited. Other risk factors that have been identified include male gender, renal dysfunction, diabetes, heart failure, corticosteroid or oral anticoagulant use, malignancy, fever within 24 hours of implantation, indwelling central venous catheter, device type, placement of more than two leads, temporary pacing, hematoma, and early reintervention.^8,10–19^

The American Heart Association (AHA), in its 2010 statement on CIED infections,^7^ and the Infectious Disease Society of America (IDSA), in its 2013 Practice Guidelines for Antimicrobial Prophylaxis in Surgery,^18^ respectively, recommend the routine administration of preprocedural antibiotics prior to CIED implantation in addition to the performance of standard-of-care aseptic techniques. Cephalosporins are preferred, as Gram-positive organisms, particularly *Staphylococcus aureus* and coagulase-negative staphylococci, are implicated in nearly two-thirds of CIED infections.^16,18^ Both the AHA and IDSA guidelines recommend a single dose of a cephalosporin such as cefazolin or cefuroxime be administered within one hour of the surgical incision.^7,18^ For patients with a ß-lactam allergy, clindamycin and vancomycin are considered acceptable alternatives; vancomycin can also be used in patients colonized with methicillin-resistant *S. aureus*. Due to longer infusion times, fluoroquinolones and vancomycin should be given within two hours of the procedure.^18^

These recommendations are supported by both retrospective and prospective studies that demonstrate a clear reduction in CIED infections with the use of preprocedural antibiotic administration.^20–25^ In a randomized, prospective trial from 1994, in which 656 patients undergoing initial pacemaker implantation received either flucloxacillin or placebo, 12 of the 13 patients who developed an infection did not receive antibiotics.^19^ In 2009, de Oliveria et al. prospectively evaluated preoperative cefazolin versus placebo in 1,000 consecutive patients undergoing CIED implantation and found a nearly 80% reduction in CIED infection with cefazolin in contrast with the placebo, leading the trial to be stopped prematurely given the clear benefit of cefazolin.^21^ Two meta-analyses have further demonstrated the utility of preprocedural antibiotics in reducing the risk of CIED infection.^23,24^

While there exist robust data supporting the use of preprocedural antibiotic prophylaxis, the use of postprocedural antibiotics remains more controversial.^26,27^ Only one known study from 1978 specifically compared postprocedural administration to periprocedural administration, where periprocedural administration was associated with a significantly lower rate of infection as compared with results in patients who received only postprocedural antibiotics (risk ratio: 0.14; 95% confidence interval: 0.03–0.60; p = 0.008).^26^ As such, while the role of preprocedural antibiotics has been established, the question remains as to whether there is added benefit from postprocedural antibiotics. Senaratne et al. reviewed patients over a 19-year period and found a protective benefit,^27^ in that CIED infection rates declined, ranging from 3.6% (no antibiotics) to 2.9% (perioperative antibiotics) and 0.4% (perioperative and postoperative antibiotics). The authors conceded, however, that changes in procedural factors over time could have accounted for improvements in the infection rates. In a prospective, multicenter study of 1,744 patients undergoing CIED replacement, 68.7% received antibiotics postoperatively in addition to receiving preoperative antibiotics. Here, there was no statistical difference in infection rates between patients who received both preoperative and postoperative antibiotics (1.4%) and those who received only preoperative antibiotics (0.9%; p = 0.582).^28^

Currently, neither the AHA nor the IDSA guidelines address the use of postprocedural antibiotics. The British Society for Antimicrobial Chemotherapy (BSAC) guidelines, however, specifically recommend against redosing of antibiotics following skin closure (level of evidence: A).^5^ Our institution lacks unique guidelines for the use of postprocedural antibiotics and, as such, they are frequently administered without consideration of the level of need. Given the paucity of data supporting the use of postprocedural antibiotics, the purpose of this study was to evaluate whether there is a role for such in infection prevention in patients undergoing CIED-related procedures. We hypothesized that there would be no further benefit with the addition of postprocedural antibiotics to routine protocols of preprocedural antibiotics and the usual perioperative sterile technique.

## Methods

The primary objective of this cohort study was to compare the rate of CIED infections in patients receiving only preoperative antibiotics with that of those who received preoperative and postoperative antibiotics. Our institution is a tertiary academic medical center that implants PPMs, ICDs, and CRT devices. The standard of care at our institution is for patients to receive preprocedural intravenous prophylactic antibiotics and local antimicrobial instillation, while the use of postprocedural antibiotic prophylaxis is done at the discretion of the operator. Patients were given cefazolin within one hour (or vancomycin within two hours) of their procedure. A majority of patients received at least one additional dose postoperatively. Local instillation technique selection depended on the implanting service; electrophysiologists predominantly used bacitracin solution, while cardiothoracic surgeons predominantly used vancomycin powder.

### Study population

**Figure 1:**
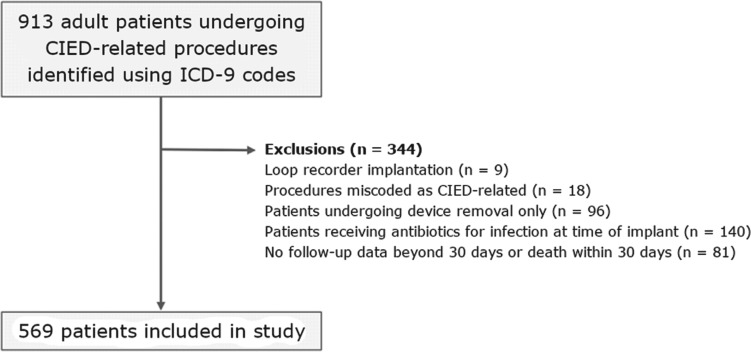
Patient selection process.

This study included a review of the data of 913 consecutive adult patients who underwent a CIED-related procedure between October 2010 and August 2014, including cases of initial implantation, revision, generator exchange, or reimplantation. Of these patients, 344 were subsequently excluded for various reasons, including due to already being treated using antibiotics for an infection and/or not participating in follow-up beyond 30 days after surgery **(Figure 1)**.

### Data collection

Patients were identified using International Classification of Diseases, ninth revision codes pertaining to CIED implantation, revision, removal, and reimplantation. Data collected included patient demographics; risk factors; procedural indication(s); device type; implanting service (ie, cardiology, cardiothoracic surgery, or pediatric cardiothoracic surgery); antibiotics; and blood culture results. Risk factors included diabetes, heart failure, liver disease, a history of malignancy, oral anticoagulant or corticosteroid use, renal dysfunction, number of prior CIED procedures, fever within 24 hours of the procedure, temporary pacing, and presence of an indwelling central venous catheter. Further chart review was performed to identify patients who later developed a CIED-related infection. Infection was defined in accordance with the BSAC guidelines, and included patients presenting with early postimplantation inflammation, uncomplicated or complicated generator pocket infection, lead infection, or CIED-associated native or prosthetic valve endocarditis.^5^

### Data analysis

Statistical analysis was performed using the Statistical Package for the Social Sciences (SPSS) version 22.0 software program (IBM Corp., Armonk, NY, USA). Patients were stratified into two groups of those who received postprocedural antibiotics and those who did not, respectively, and each group was compared in terms of risk factors. The chi-squared and Fisher’s exact tests were used for categorical variables. After univariate analysis was performed to identify clinically significant risk factors, multivariable logistic regression analysis was employed to detect any independent effects. Due to the low incidence of infection, multivariable analysis was limited to considering three risk factors.

A statistical analysis of the data was conducted using SPSS version 15 (IBM Corp., Armonk, NY, USA). Normally distributed descriptive variables were expressed as means ± standard deviations; variables with skewed distribution were expressed as medians and ranges (minimum to maximum). Patients with PPM/ICD infections and controls were compared with regard to risk factors. Pocket infection and systemic infection subgroups were also compared. The chi-squared and Fisher’s exact tests were used for categorical variables. The Student’s t-test was used for normally distributed continuous numeric variables. The Mann–Whitney U test was used for the comparison of continuous numeric variables with skewed distribution. In order to evaluate the independent effects of clinically significant risk factors (eg, age, gender) and those that had a significant effect on outcome based on univariate analysis (p < 0.05), multivariate logistic regression analysis was performed. The statistical significance level was considered as p < 0.05.

## Results

Patients were stratified and analyzed in two groups, those who received postprocedural antibiotics and those who did not. Overall, approximately 70.5% of patients received postoperative antibiotic prophylaxis. A comparison of baseline characteristics between these two groups is shown in **Table 1**. There were no significant differences in terms of baseline patient characteristics including the prevalence of diabetes, heart failure, renal impairment, or steroid and anticoagulant use. There were several differences in terms of periprocedural factors between the two groups, including a higher incidence of fever (5% versus 0.4%; p = 0.008) and periprocedural pacing (14.4% versus 4.3%; p = 0.001) in patients not receiving antibiotics, while there was a higher percentage of patients receiving more than two new implanted leads among the patients who received postoperative antibiotics (64.3% versus 52%; p = 0.005). Certain data were consistently missing in approximately half of the sample population, including preoperative vital signs (fever), the presence of an indwelling central venous line, the use of periprocedural pacing, and postoperative physical examination findings (for recording the presence or absence of hematoma).

A notable difference between the postprocedural antibiotic and nonantibiotic groups was the performing service, reflecting an institution-wide tendency of electrophysiologists to routinely prescribe postprocedural prophylaxis but an avoidance of the same by cardiothoracic surgeons. Nearly 95% of patients who received postoperative antibiotics had their CIEDs implanted by an electrophysiologist as compared with approximately 41% of the group not receiving antibiotics **(Table 1)**. Those in the postoperative antibiotic group were more likely to have undergone an initial implantation by an electrophysiologist, while those who did not receive postoperative antibiotics were more evenly split between the electrophysiology and cardiothoracic surgery services. In the entire cohort, approximately 80% of CIED implants were performed by electrophysiologists.

The observation of a lower incidence of postoperative hematoma despite no significant difference in anticoagulant use between the groups as well as reintervention within 90 days in the group receiving postoperative antibiotics may suggest the postoperative antibiotic group includes a lower-risk group of patients. These differences could reflect higher-risk patients being more likely to be referred to a cardiothoracic surgeon as opposed to an electrophysiologist.

**Figure 2:**
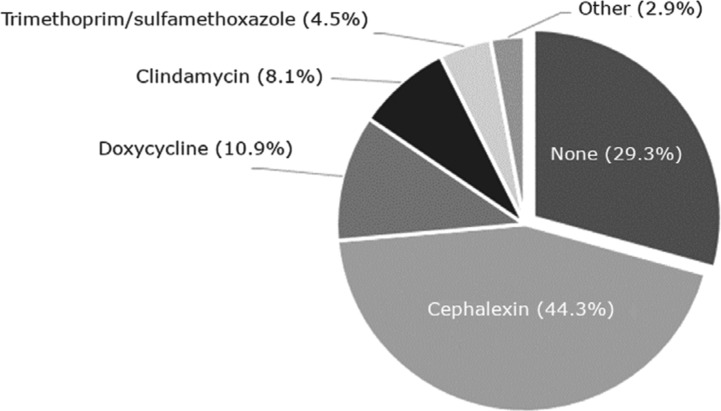
Prescribed postoperative antibiotics.

Regarding antimicrobial use, the majority of CIED procedures completed at our institution were supplemented with postoperative antibiotics. Cephalexin was the most commonly prescribed antibiotic at a rate of 44.3% and appears to be the drug of choice for the electrophysiology service line, although the rationale for each individual antibiotic choice was not documented **(Figure 2)**. In decreasing order of frequency, doxycycline, clindamycin, and trimethoprim/sulfamethoxazole combined composed antibiotic use in approximately 24% of the total population. Infrequently miscellaneous antibiotics such as cefdinir, ciprofloxacin, cefazolin, ceftriaxone, and vancomycin were used as postprocedural prophylaxis. At least two patients definitively developed allergies to the prophylactic cephalexin, although it was difficult to determine from chart review alone as to whether there were any other adverse reactions.

Duration of postoperative prophylaxis ranged from one to 14 days, with 87.3% of patients receiving between three and five days of antibiotics. One patient, designated as at a high risk for infection due to prior methicillin-resistant *S. aureus* bacteremia from an infected hemodialysis fistula, received 14 days of vancomycin for prophylaxis following initial placement of a single-chamber pacemaker. Another patient with an existing ICD received 14 days of cephalexin prophylaxis, having undergone failed revision by an electrophysiologist and removal and replacement by a cardiothoracic surgeon, which was subsequently followed by another revision. Among the 569 patients included in this study, 29 (5.1%) ultimately developed CIED infections **(Table 2)**.

There was no statistically significant difference in the incidence of infection between patients who received postoperative antibiotics and those who did not (4.5% versus 6.1%; p = 0.398). Only two patients developed systemic infections, both of whom had received postoperative antibiotics, although this was not a statistically significant finding due to the small incidence.

Several previously-identified risk factors for CIED infection were found to be statistically significant upon univariate analysis, including heart failure, chronic liver disease, and anticoagulant use **(Table 3)**. Incomplete documentation, as previously mentioned, significantly reduced the number of patients able to be included in the analysis for any individual risk factor, and may have contributed to a lack of statistical significance for more variables.

Due to the low incidence of infection, multivariable analysis **(Table 4)** was limited to considering three risk factors. When the use of postoperative antibiotics was analyzed with the two most significant risk factors by univariate analysis (heart failure and chronic liver disease), it was still not deemed a statistically significant risk factor for infection, although it trended toward being protective (adjusted odds ratio: 0.692; 95% confidence interval: 0.314–1.525; p = 0.361).

## Discussion

National guidelines supported by primary literature and meta-analyses currently recommend the use of preprocedural antibiotics to prevent infectious complications following CIED implantation.^5,7^ Conversely, there is limited evidence to support postoperative antibiotic use, and the decision appears to be based on physician preference and/or possibly on a subjective assessment of patient risk, as is true at our institution. We hypothesized that there would be no added benefit from the addition of postprocedural antibiotics and, in the present study, failed to find a large difference in CIED infection rates between patients who received postoperative antibiotics and those who did not. There was, however, a trend toward a lower infection risk with the use of postoperative antibiotics. The overall rate of CIED infection was 5.1%, which is consistent with findings in prior studies, although somewhat higher than more recent estimates of 0.5% to 2.2%.^8^

Our results conflict with those of Senaratne et al., who demonstrated a significant protective effect from the use of postoperative antibiotics.^27^ In their study, patients were retrospectively reviewed over a 19-year period and separated into different cohorts depending on the CIED antibiotic prophylaxis strategy of the time. It should be noted that infection rates could have differed in part due to time-specific practices (ie, because the study period ranged from 1992 to 2008) rather than the implementation of postoperative antibiotic use.

Our study is not without limitations. As a retrospective review, the validity of the presented results is contingent on the accurate documentation and charting of important events. During the study period, our institution transitioned from paper charts to an electronic medical records system. We noted more consistent postoperative antibiotic prescribing practices following this transition and, more recently, with the implementation of order sets that prompt clinicians to order antibiotic therapy. We also could not control for patients presenting to outside hospitals for complications due to their implanted CIEDs, although charts were reviewed outside of the 90-day threshold to verify the presence or absence of a CIED infection. As a referral center for a large portion of South Carolina, many patients who underwent implantation at our institution only followed up with their local cardiologist; therefore, those patients without follow-up at our institution were also excluded.

Among our cohort, we were unable to control for the unequal distribution of patients between the two groups, as electrophysiologists preferentially prescribed postoperative antibiotics and cardiothoracic surgeons generally did not. Therefore, our results must be interpreted with caution, as it is difficult to control for differences in operative technique between CIED implantations performed by electrophysiologists and cardiothoracic surgeons, respectively, as well as the intrinsic differences in individual patient risk between those referred to cardiology as opposed to cardiothoracic surgery. However, the resulting sample bias, in which patients who received antibiotics were likely to be at lower risk in comparison with those who did not receive antibiotics, should have instead favored the use of antibiotics. Finally, our study was underpowered to detect a true impact of antibiotics on the rate of CIED infection. It should be noted that a sample size of 6,200 patients would be needed to detect a statistical difference between groups, which is not considered to be feasibly obtainable from an electronic health records database at a single institution within a reasonable time period.

Outside of traditional intravenous or oral antibiotics, other strategies can be employed to reduce the risk of infection. Data have been favorable regarding the use of antibiotic-impregnated dissolvable pouches in the reduction of CIED infection.^29^ In another recent study, Manolis et al. reported a protocol involving a preparation of the skin with alcohol and povidone iodine as well as the use of preoperative and postoperative antibiotics for a total duration of four to five days. Their results included an infection rate of 0.26% at a mean follow-up point of more than two years, which is lower than the rates reported in older studies.^30^ Although the intent of the trial by Manoulis et al. was not to evaluate the utility of postoperative antibiotics, it underscored the need for randomized, controlled data comparing different prophylactic antibiotic strategies. The recently reported Prevention of Arrhythmia Device Infection Trial (PADIT), which was published following the conclusion of our study, provides strong evidence that an incremental approach of preprocedural, intraprocedural, and postprocedural antibiotic therapy did not improve device infection outcomes versus preprocedural antibiotics alone.^31^ Furthermore, patients prespecified to be high risk for device infection showed no difference between the aforementioned antibiotic groups. These data, in concert with our findings, illustrate that a strategy involving more antibiotic therapy is unlikely to be advantageous in reducing the infectious complications related to CIED implantation.

Our data, though retrospective and underpowered, have revealed several concerning clinical practices at our own institution, highlighting the need for future studies and clearer guidelines. With regard to antibiotic prescribing practices at our institution, there was no consensus about drug choice or duration. Generally, there was no documented rationale for drug choice, although, in a few cases, a high infection risk was cited as the reason for a longer duration of prophylaxis. Multiple patients received seven or more days of prophylactic antibiotics. A separate small study of 178 patients undergoing PPM implantation randomized the patients to receive either a short (48-hour) course of antibiotics or a longer (seven-day) course. Both groups received the same antibiotic regimen, and there was no statistical difference in infection rates between the two groups.^32^ The optimal duration of prophylactic antibiotics has otherwise not been specifically evaluated.

Clinicians at our institution prescribed nine different antibiotics for postoperative prophylaxis. Guidelines for perioperative prophylaxis recommend antibiotics that cover Gram-positive cocci, particularly *Staphylococcus* species. Broader coverage with fluoroquinolones and second- and third-generation cephalosporins is likely unnecessary. Numerous patients received multiple antibiotics concurrently, presumably because the intravenous preoperative antibiotic was not discontinued before the oral postoperative antibiotic was ordered. Although excluded from our study, we noted that many patients with an active infection were prescribed additional postoperative antibiotics, despite already receiving broad-spectrum antibiotics or antibiotics in the same drug class. This excess use of antibiotic exposes the patient to an unnecessary risk of resistance, allergic reaction, or other adverse effects.

## Conclusion

Our findings provide additional support for the suggestion that it may be reasonable to withhold the prescription of postoperative antibiotics in many patients presenting for CIED implantation. Reducing instances of unnecessary administration of antibiotics may provide opportunities to lower patient costs, avoid antibiotic-mediated adverse drug reactions, and potentially limit the risk of antimicrobial resistance. However, an individualized risk factor evaluation of patient comorbidities and procedural characteristics may be needed for each case to aid in determining whether postoperative antibiotics are reasonable in specific patients. Other important infection prevention strategies—such as the avoidance of temporary pacing, delaying procedures in those with active infection, pursing the appropriate preoperative withdrawal of antithrombotic therapy, and ensuring appropriate skin preparation—should also be incorporated into the standard of care at an institutional level.

## Figures and Tables

**Table 1:** Comparison of Baseline Characteristics Between Patients Who Received Postoperative Antibiotic Prophylaxis and Those Who Did Not

	Postoperative Antibiotics (n = 401), n (%)	No Postoperative Antibiotics (n = 168), n (%)	p-value
Patient characteristics			
Male gender	256 (63.8)	104 (58.1)	0.188
Renal impairment^*^	165 (41.3)	62 (35)	0.158
Dialysis	8 (2)	6 (3.4)	0.381
Heart failure	190 (52.6)	92 (51.4)	0.786
Chronic liver disease	18 (4.5)	5 (2.8)	0.331
Diabetes	122 (30.4)	56 (31.3)	0.835
History of malignancy	62 (15.5)	23 (12.8)	0.411
Anticoagulation	147 (36.7)	64 (35.8)	0.834
Chronic steroids	15 (3.8)	7 (3.9)	0.926
Periprocedural factors			
Fever^**^	1 (0.4)	5 (5)	0.008***
Prior CIED infection	8 (2)	6 (3.4)	0.381
Indwelling line	15 (6.7)	11 (11.7)	0.140
Periprocedural pacing	11 (4.3)	13 (14.4)	0.001***
Two or more new leads implanted	258 (64.3)	93 (52)	0.005***
Device			0.518
ICD	149 (37.2)	59 (33)	
PPM	156 (38.9)	72 (40.2)	
Combination	2 (0.5)	0 (0)	
CRT-D	83 (20.7)	45 (25.1)	
CRT-P	11 (2.7)	3 (1.7)	
Performing service			< 0.001***
Cardiology	380 (94.8)	74 (41.3)	
Cardiothoracic surgery	17 (4.2)	88 (49.2)	
Pediatric cardiology	2 (0.5)	14 (7.8)	
Combined	2 (0.5)	3 (1.7)	
Procedure			< 0.001***
Initial	299 (74.6)	82 (45.8)	
Revision	87 (21.7)	94 (52.5)	
Reimplantation	14 (3.5)	3 (1.7)	
Postoperative hematoma	25 (9.9)	19 (17.3)	0.047***
Reintervention within 90 days	16 (4)	15 (8.4)	0.031***

CIED: cardiovascular implantable electronic device; CRT-D: cardiac resynchronization therapy defibrillator; CRT-P: cardiac resynchronization therapy pacemaker; ICD: implantable cardioverter-defibrillator; PPM: permanent pacemaker.

*Serum creatinine ≥ 1.5 mg/dL or estimated creatinine clearance < 60 mL/min upon admission.

**Fever within 24 hours prior to the procedure (defined as greater than 38°C) or hypothermia (defined as less than 35°C).

***Statistically significant.

**Table 2:** Incidence of CIED Infection According to the Presence or Absence of Postoperative Antibiotics

	Postoperative Antibiotics (n = 401), n (%)	No Postoperative Antibiotics (n = 168), n (%)	p-value
Any infection	18 (4.5)	11 (6.1)	0.398
Systemic infection	2 (0.5)	0 (0)	1.000

CIED: cardiovascular implantable electronic device.

**Table 3:** Risk Factors for CIED Infection by Univariate Logistic Regression

Previously Identified Risk Factors^10–14^	CIED Infection	
	Odds Ratio (95% Confidence Interval)	p-value
Patient characteristics		
Renal impairment	1.357 (0.633–2.908)	0.433
Dialysis	3.327 (0.709–15.614)	0.128
Heart failure	2.504 (1.090–5.750)	0.030*
Chronic liver disease	6.157 (2.108–17.985)	0.001*
Diabetes	1.901 (0.894–4.041)	0.095
Anticoagulation	2.247 (1.059–4.768)	0.035*
Chronic steroids	3.225 (0.897–11.595)	0.073
Prior CIED infection	1.478 (0.187–11.703)	0.711
Periprocedural factors		
Indwelling line	0.542 (0.070–4.209)	0.558
Periprocedural pacing	1.199 (0.152–9.445)	0.863
Two or more new leads implanted	1.253 (0.572–2.746)	0.573
Prior CIED procedure	0.578 (0.273–1.222)	0.151
Postoperative antibiotics	0.718 (0.332–1.553)	0.400

CIED: cardiovascular implantable electronic device.

*Statistically significant.

**Table 4:** Risk Factors for CIED Infection According to Multivariable Logistic Regression

Risk Factor	CIED Infection	
	Adjusted Odds Ratio (95% Confidence Interval)	p-value
Heart failure	2.533 (1.092–5.974)	0.030*
Chronic liver disease	6.502 (2.168–19.398)	0.001*
Postoperative antibiotics	0.692 (0.314–1.525)	0.361

CIED: cardiovascular implantable electronic device.

*Statistically significant.
